# An Alternative Operative Approach to Lumbar Spondylolisthesis

**DOI:** 10.7759/cureus.25276

**Published:** 2022-05-24

**Authors:** Matthew Putty, Gina Guglielmi, Hamad Farhat

**Affiliations:** 1 Neurosurgery, Advocate Christ Medical Center, Oak Lawn, USA; 2 Neurosurgery, Carle Foundation, Champaign, USA

**Keywords:** anterior lumbar spine surgery, anterior lumbar, high grade l5-s1 spondylolisthesis, spondylolisthesis, lumbar-fusion

## Abstract

Lumbosacral spondylolisthesis is a frequently encountered pathology with high-grade spondylolisthesis being the least common. A circumferential construct is usually the preferred treatment as these can resist the shearing forces present at L5-S1. However, the severity of the slip, sacral inclination, and the slip angle may make a traditional anterior approach difficult to achieve. In this case series, we present three patients with axial back pain that were treated with an anterior L5-S1 transvertebral cage. This technique is intended for both grade II spondylolisthesis and high sacral slope. The L5-S1 transvertebral cage may be sufficient to prevent further listhesis, fuse the patient, and alleviate axial back pain.

## Introduction

Adult degenerative lumbosacral spondylolisthesis is a commonly encountered pathology, with a small percentage of these patients progressing towards high-grade spondylolisthesis [1]. The main goals of surgical management for degenerative lumbosacral spondylolisthesis are pain relief and resolution of neurological deficits, if present [1,2]. Typically, this is accomplished by decompression and fusion [1,2]. However, the intraoperative management of this condition is often complicated by the anatomical distortion caused by the L5 vertebral body over S1, a high degree of sacral inclination, and a high sacral slope [1]. 

There are multiple surgical approaches described on how to address this pathology and most describe a three-column construct or circumferential fusion [1-6]. Authors have reported that circumferential fusion produces better long-term results than either isolated posterior or anterior fusion [2]. The severity of the slip of L5 on S1, with a high degree of sacral inclination and sacral slop, makes a traditional anterior interbody fusion very difficult or impossible. A few techniques described have included: transdiscal pedicle screw fixation, anterior or posterior transvertebral fibular graft fusion, or posterior transvertebral cage fixation [3-6]. Oblique lumbar interbody fusion is another option but the anatomy vascular anatomy variations distal to the bifurcation of the great vessels can limit this technique [7]. Traditional transforaminal lumbar interbody fusion and posterior lumbar fusion have lower failure rates for grade II spondylolisthesis, not to mention higher rates of blood loss and longer length of hospital stay [1,2]. One approach described an anterior lumbar interbody fusion at L4-L5 with the implementation of an in-situ transvertebral cage at L5-S1 with posterior L5-S1 transdiscal screws [1]. 

Here, we present a small case series where we describe another approach for grade 2 lumbosacral spondylolisthesis with axial back pain. This approach utilizes the access of an anterior lumbar interbody with the use of a cage traversing the L5 vertebral body, L5-S1 disc space, and the S1 vertebral body. This technique permits the surgeon to not have to address the steep angle of the L5-S1 disc space that is present in high sacral slopes and grade 2 spondylolisthesis.

## Case presentation

Surgical technique

**Figure 1:**
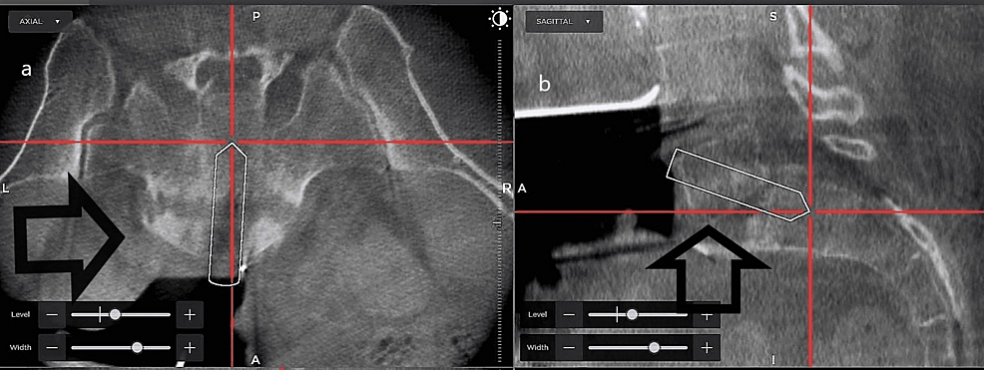
The trajectory of the transvertebral cage is laid out. Panel (a) is showing an axial image of the cage trajectory traversing the L5-S1 disc space as depicted by the arrow. Panel (b) is showing the cage trajectory entering the anterior superior corner of the L5 vertebral body, through the L5-S1 disc space and into the S1 vertebral body.

The patient is positioned supine on a radiolucent Jackson table. The abdomen is then prepared and draped in a sterile manner. A one-centimeter incision is made over the right anterior superior iliac spine (ASIS) and a navigation pin is placed in the ASIS. A general surgeon then performs a retroperitoneal exposure of the L5 vertebral body and L5-S1 disc space. Once the disc space is exposed, fluoroscopy is used to confirm the correct location. Next, the O-arm navigation system is brought into the operating room and a spin is conducted. Using O-arm navigation and the spinal software used for O-arm navigation, the entry point of the transvertebral cage is planned, which frequently is the anterior, superior edge of the L5 vertebral body, as depicted in Figure 1.

**Figure 2:**
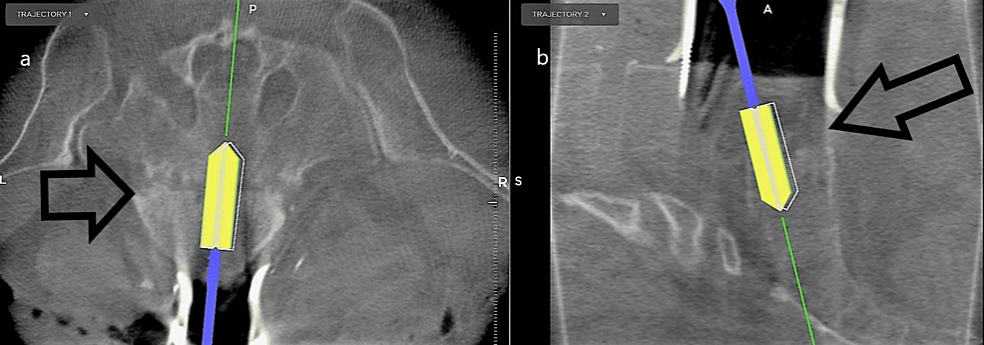
The trajectory is tapped using a tap on neuro-navigation. Panel (a) is depicting an axial view of the tap traversing the L5-S1 disc space. Panel (b) is depicting a sagittal view of the tap with an image projecting the width of the cage.

The trajectory is then drilled out and then the tract is tapped with stealth navigation, as shown in Figure 2.

**Figure 3:**
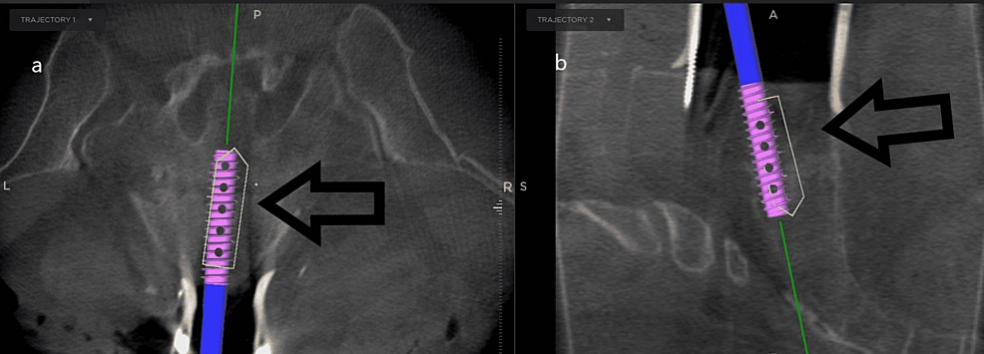
Panel (a) is showing the transvertebral cage being inserted under stealth navigation using axial imaging. Panel (b) is showing the cage being inserted through a sagittal window.

The cage is prepared with demineralized bone matrix and bone morphogenic protein. Then the cage is placed with stealth guidance through the L5 vertebral body, the L5-S1 disc space into the S1 body, as depicted in Figure 3.

Fluoroscopy is then employed to obtain lateral and anterior-posterior shots to confirm the satisfactory placement of the cage. Then the field is copiously irrigated, and the access surgeon closes the patient.

Case 1

The first patient is a 48-year-old female with bilateral pars defects at L5 with resultant grade 2 spondylolisthesis presenting with chronic axial back pain. The patient denied any symptoms of neurogenic claudication or radicular complaints. She had attempted conservative management with minimal relief. She underwent an anterior lumbar interbody fusion with a transvertebral cage at L5-S1. In the immediate postoperative period, she described right anterior thigh paresthesia, but no other complaints. Her back pain had improved and she was discharged on postoperative day two. On the two-week visit, her thigh paresthesia was still present, but improved, and her axial back pain was also improved. At her six-week follow-up, her right thigh paresthesia had resolved, her axial back pain was improved and she had flexion-extension x-rays demonstrating no active listhesis.

Case 2

The second patient is a 50-year-old male who had a prior L5-S1 posterolateral fusion, instrumentation, and L5 laminectomy ten years prior. He presented with axial back pain as well as neurogenic claudication and left L5 radiculopathy. Computed tomography showed bilateral pars defects at L5 with a grade 2 spondylolisthesis of L5-S1. He underwent an anterior lumbar interbody fusion with a transvertebral cage at L5-S1. He was in the hospital for three days after the procedure with no acute neurological issues. At his two-week postoperative visit, he reported his back pain and radicular pain were improved but was noted to have a superficial infection. He was on oral antibiotics for 10 days to treat the infection. At the six-week follow-up, he reported improvement in his back pain and his ability to ambulate.

Case 3

The final patient is a 45-year-old male who presented with worsening axial back pain as well as neurogenic claudication. He tried epidural steroid injections and physical therapy with minimal relief of his symptoms. Computed tomography showed a bilateral L5 pars defect with a grade 2 spondylolisthesis of L5 on S1. He then underwent an anterior lumbar interbody fusion with an L5-S1 transvertebral cage. He did well postoperatively and was discharged on postoperative day one. At his two-week follow-up, he reported his back pain had resolved and he could walk much further now. At his six-week follow-up visit, he continued to do well with no axial back pain or neurogenic claudication. He had lumbar flexion-extension that did not show progression of his listhesis.

## Discussion

The rate of pseudoarthrosis in adolescents with pedicle instrumentation with posterolateral fusion from the transverse process down to the sacral ala is reported to be 17-40% [8-10]. The failure rate behind pedicle instrumentation with posterolateral fusion in high-grade spondylolisthesis is postulated to be multifactorial. First, the L5 transverse process will be deep and more difficult to access but will often be small or even dysplastic leading to less bony surface area to permit a fusion down to the sacral ala [6,11]. Second, without an anterior axial load sharing, the axial loading forces will transmit through the pedicle screws, which is postulated to result in micromotion of the instrumented construct that can lead to hardware failure and inhibit fusion. Essentially, pedicle screws and rod construct are mechanically disadvantaged to handle the strong shear forces between L5-S1 in higher-grade spondylolisthesis. For these reasons, it seems paramount to have an anterior component in the construct to help with the anterior axial load sharing [11-14].

Over the years, several approaches have been described on how to add anterior structural support in high-grade spondylolisthesis [1,3,9,15,16]. In 1994, Abdu et al., described a technique where posterior sacral pedicle screws were inserted and designed to cross the L5-S1 disc space into the L5 vertebral body [3]. The idea was that these multicortical screws would provide additional strength anteriorly to handle the axial loading forces, thus reducing the high shear forces experienced at the lumbosacral junction [15,16]. By reducing the shear forces, this could potentially reduce or eliminate the micromotion of the construct thus permitting fusion and minimizing construct failure. It was reported, in a cadaveric model, that transdiscal L5-S1 screw fixation produced a construct that was 1.6-1.8 stiffer than traditional pedicle screw instrumentation [17]. The authors of this paper even found that the transdiscal fixation was equal to that of an interbody fusion with pedicle screw instrumentation [17]. 

The transdiscal screw fixation set the stage for the next evolution, posterior transvertebral interbody fusion with an autograft-filled 7-millimeter cage, which was described by Bartolozzi and coauthors in 2003 [4]. An autograft-filled titanium mesh cage immediately provides structural stability in the anterior column, with a higher elasticity modulus to resist shear forces across the L5-S1 disc space, in contrast with fibular autograft or allograft [4,18]. Furthermore, the resistance to shear force increases with the radius of the cage squared, such that a 12-millimeter cage is nearly twice as resistant to shear forces as a 7-millimeter cage [1].

Beringer et al. described another technique where they treated high-grade spondylolisthesis with an L4-L5 anterior lumbar interbody fusion, an anterior L5-S1 transvertebral cage, and L4-S1 pedicle screws with the S1 pedicle screws being transdiscal at L5-S1 [1]. With the addition of a posterior decompression, they reported positive excellent clinical results without neurological complications. We propose that in patients with higher-grade spondylolisthesis who have axial back pain, an L5-S1 transvertebral cage may be enough to prevent further slippage, fuse them, and ameliorate mechanical back pain. The transvertebral cage is produced by Medtronic (Minneapolis, Minnesota, United States), and is originally indicated for sacroiliac joint fusions. The cage width is 12-millimeters, allowing for more resistance to shear forces, and can be filled with demineralized bone matrix and bone morphogenic protein to allow for fusion. This cage can be placed under navigation, which increases the accuracy of placement.

Our case series, though small, has shown good results with alleviation in the patients' complaints as well as stable lumbar flexion-extension x-rays. Unfortunately, we do not have long-term follow-up to demonstrate if fusion occurred at the L5-S1 level and this would be the next step in determining the usefulness of the approach. There are limitations to this approach, with the main one being there is no disc preparation performed, which could potentially limit the rate of fusion across this level. Without long-term follow-up, both the rate of fusion and pseudoarthrosis is unknown and it is possible that the patient may need posterior instrumentation.

## Conclusions

There may be a role for an L5-S1 transvertebral cage in the treatment of grade 2 L5-S1 anterolisthesis and high-grade sacral slope in cases where traditional anterior lumbar interbody fusion is difficult. In patients with a high-grade spondylolisthesis, this technique can be used as a stand-alone construct performed in a single surgery. The surgery is efficient, requires less exposure than a standard anterior lumbar interbody, and is compatible with navigation.
